# Survival benefit of ixazomib, lenalidomide and dexamethasone (IRD) over lenalidomide and dexamethasone (Rd) in relapsed and refractory multiple myeloma patients in routine clinical practice

**DOI:** 10.1186/s12885-020-07732-1

**Published:** 2021-01-15

**Authors:** Jiri Minarik, Tomas Pika, Jakub Radocha, Alexandra Jungova, Jan Straub, Tomas Jelinek, Ludek Pour, Petr Pavlicek, Martin Mistrik, Lucie Brozova, Petra Krhovska, Katerina Machalkova, Pavel Jindra, Ivan Spicka, Hana Plonkova, Martin Stork, Jaroslav Bacovsky, Lenka Capkova, Michal Sykora, Petr Kessler, Lukas Stejskal, Adriana Heindorfer, Jana Ullrychova, Tomas Skacel, Vladimir Maisnar, Roman Hajek

**Affiliations:** 1 Department of Hemato-Oncology, Faculty of Medicine and Dentistry, Palacky University Olomouc and University Hospital Olomouc, Olomouc, Czech Republic; 2 4th Department of Internal Medicine – Hematology, Faculty Hospital and Charles University in Hradec Kralove, Hradec Kralove, Czech Republic; 3 Hematology and Oncology Department, Charles University Hospital Pilsen, Pilsen, Czech Republic; 4 1st Medical Department – Clinical Department of Haematology, First Faculty of Medicine and General Teaching Hospital Charles University, Prague, Czech Republic; 5 Department of Hematooncology, University Hospital Ostrava and Faculty of Medicine University of Ostrava, Ostrava, Czech Republic; 6 Department of Internal Medicine, Hematology and Oncology, University Hospital Brno and Faculty of Medicine Masaryk University, Brno, Czech Republic; 7 Department of Internal Medicine and Hematology, 3rd Faculty of Medicine, Charles University and Faculty Hospital Kralovske Vinohrady, Prague, Czech Republic; 8 Department of Hematology and Transfusiology, Faculty of Medicine, University Hospital Bratislava, Bratislava, Slovakia; 9 Institute of Biostatistics and Analyses, Ltd., Brno, Czech Republic; 10 Department of Clinical Hematology, Hospital Ceske Budejovice, Ceske Budejovice, Czech Republic; 11 Department of Hematology and Transfusion Medicine, Hospital Pelhrimov, Pelhrimov, Czech Republic; 12 Department of Hematology, Silesian Hospital in Opava, Opava, Czech Republic; 13 Department of Hematology, Hospital Liberec, Liberec, Czech Republic; 14 Department of Clinical Hematology, Regional Health Corporation, Masaryk Hospital in Usti nad Labem, Usti nad Labem, Czech Republic; 15 1st Department of Medicine, First Faculty of Medicine, Charles University and General Hospital in Prague, Prague, Czech Republic; 16 Millennium Pharmaceuticals, Inc., a wholly owned subsidiary of Takeda Pharmaceutical Company Limited, Cambridge, MA USA

**Keywords:** Multiple myeloma, Ixazomib, Lenalidomide, Dexamethasone, Clinical trial, Patient registry

## Abstract

**Background:**

We have performed a head to head comparison of all-oral triplet combination of ixazomib, lenalidomide and dexamethasone (IRD) versus lenalidomide and dexamethasone (RD) in patients with relapsed and refractory multiple myeloma (RRMM) in the routine clinical practice.

**Methods:**

A total of 344 patients treated with IRD (*N* = 127) or RD (*N* = 217) were selected for analysis from the Czech Registry of Monoclonal Gammopathies (RMG). Descriptive statistics were used to assess patient’s characteristics associated with the respective therapy. The primary endpoint was progression free survival (PFS), secondary end points included response rates and overall survival (OS). Survival endpoints were plotted using Kaplan-Meier methodology at 95% Greenwood confidence interval. Univariable and multivariable Cox proportional hazards models were used to evaluate the effect of treatment regimens and the significance of uneven variables. Statistical tests were performed at significance level 0.05.

**Results:**

In the whole cohort, median PFS for IRD was 17.5 and for RD was 11.5 months favoring the all-oral triplet, *p* = 0.005; in patients within relapse 1–3, the median PFS was 23.1 vs 11.6 months, *p* = 0.001. The hazard ratio for PFS was 0.67 (95% confidence interval [CI] 0.51–0.89, *p* = 0.006). The PFS advantage translated into improved OS for patients treated with IRD, median 36.6 months vs 26.0 months (*p* = 0.008). The overall response rate (ORR) was 73.0% in the IRD group vs 66.2% in the RD group with a complete response rate (CR) of 11.1% vs 8.8%, and very good partial response (VGPR) 22.2% vs 13.9%, IRD vs RD respectively. The IRD regimen was most beneficial in patients ≤75 years with ISS I, II, and in the first and second relapse. Patients with the presence of extramedullary disease did not benefit from IRD treatment (median PFS 6.5 months). Both regimens were well tolerated, and the incidence of total as well as grade 3/4 toxicities was comparable.

**Conclusions:**

Our analysis confirms the results of the TOURMALINE-MM1 study and shows benefit of all-oral triplet IRD treatment versus RD doublet. It demonstrates that the addition of ixazomib to RD improves key survival endpoints in patients with RRMM in a routine clinical setting.

## Background

The novel triplet IRD (ixazomib, lenalidomide, dexamethasone) has been approved in more than 70 countries for patients with relapsed and refractory multiple myeloma (RRMM) based on the results of several clinical trials including TOURMALINE-MM1 (NCT01564537) [1–8].

The results of clinical trials, however, are usually not representative of the typical “real-world” patient in the community setting; the population recruited to these studies is subject to significant selection bias which utilizes inclusion and exclusion criteria to predominantly enroll less pre-treated and healthier patients, usually with better overall life expectancy [9, 10].

This prospectivaly defined analysis of the Czech Registry of Monoclonal Gammopathies (RMG) will present real-world clinical outcomes for patients with RRMM treated in routine clinical practice in the Czech Republic. We directly compared patients who were treated with the IRD triplet to those who were treated with a doublet (RD: lenalidomide, dexamethasone), and present further analysis comparing these real-world outcomes to the reported results of the TOURMALINE-MM1 clinical trial.

## Methods

### Patient population

We analysed a cohort of 344 patients with RRMM treated between 2016 and 2018 with IRD (*N* = 127) or RD (*N* = 217). IRD was administered as a part of the Named Patient Program (NPP), and all data were collected in the RMG. All patients were treated outside the context of a clinical trial.

### Study design

Patients were unselected by any clinical criteria, and treatment with IRD was determined only by the local availability of the NPP for ixazomib. The demographic and clinical characteristics of IRD and RD cohorts is in Table 1. We excluded patients treated within the first line of therapy, patients with missing data for primary endpoints, patients on clinical trials, and patients who switched treatment to another combination regimen.

**Table 1:** Baseline characteristics

	IRD (***N*** = 127)	RD (***N*** = 217)	***p***^**1**^
**Sex**, n (%)	*n* = 127	*n* = 217	
Male	72 (56.7%)	107 (49.3%)	0.219
Female	55 (43.3%)	110 (50.7%)	
**Age (at treatment initiation)**, n (%)	*n* = 127	*n* = 217	
≤ 65	57 (44.9%)	80 (36.9%)	**0.004**
66–75	59 (46.5%)	89 (41.0%)	
> 75	11 (8.7%)	48 (22.1%)	
Median (min–max)	66.0 (41.0–84.0)	68.0 (41.0–90.0)	**0.002**
**M-protein type**, n (%)	*n* = 126	*n* = 217	
IgG	82 (65.1%)	132 (60.8%)	0.470
IgA	22 (17.5%)	37 (17.1%)	
LC only	18 (14.3%)	42 (19.4%)	
Other^a^	4 (3.2%)	6 (2.8%)	
**Light chain type**, n (%)	*n* = 126	*n* = 216	
Kappa	78 (61.9%)	142 (65.7%)	0.122
Lambda	44 (34.9%)	73 (33.8%)	
Biclonal	4 (3.2%)	1 (0.5%)	
**ISS stage (at treatment initiation)**, n (%)	*n* = 108	*n* = 175	
Stage 1	51 (47.2%)	63 (36.0%)	0.136
Stage 2	28 (25.9%)	62 (35.4%)	
Stage 3	29 (26.9%)	50 (28.6%)	
**ECOG performance status**, n (%)	*n* = 124	*n* = 197	
0	19 (15.3%)	22 (11.2%)	0.093
1	84 (67.7%)	120 (60.9%)	
2	19 (15.3%)	44 (22.3%)	
3–4	2 (1.6%)	11 (5.6%)	
**Creatinine level (umol/l)**, n (%)	*n* = 126	*n* = 205	
≤ 176	118 (93.7%)	179 (82.5%)	0.092
> 176	8 (6.3%)	26 (12.0%)	
Median (min–max)	88.0 (31.0–668.0)	90.0 (45.0–902.0)	0.340
**Extramedullary mass**, n (%)	*n* = 127	*n* = 210	
No	109 (85.8%)	196 (93.3%)	**0.034**
Yes	18 (14.2%)	14 (6.7%)	
**Number of previous lines of treatment**, n (%)	*n* = 127	*n* = 217	
1	73 (57.5%)	123 (56.7%)	0.823
2	30 (23.6%)	54 (24.9%)	
3	11 (8.7%)	23 (10.6%)	
4	8 (6.3%)	8 (3.7%)	
≥ 5	5 (3.9%)	9 (4.1%)	
Median (min–max)	1.0 (1.0–9.0)	1.0 (1.0–7.0)	0.979
**Previous treatment**, n (%)	*n* = 127	*n* = 217	
ASCT	79 (62.0%)	94 (43.3%)	**< 0.001**
PI (bortezomib or carfilzomib)	123 (96.9%)	198 (91.2%)	**0.047**
Bortezomib	120 (94.5%)	195 (89.9%)	0.162
Carfilzomib	6 (4.7%)	6 (2.8%)	0.371
IMiD (lenalidomide or thalidomide or pomalidomide)	64 (50.4%)	120 (55.3%)	0.433
Lenalidomide	22 (17.3%)	33 (15.2%)	0.648
Thalidomide	55 (43.3%)	105 (48.4%)	0.373
Pomalidomide	2 (1.6%)	–	0.136
**Disease status**, n (%)	*n* = 120	*n* = 193	
Relapsed	95 (79.2%)	134 (69.4%)	0.067
Refractory	25 (20.8%)	59 (30.6%)	
➣ Primary refractory	10 (40.0%)	25 (42.4%)	1.000
➣ Relapsed and refractory	15 (60.0%)	34 (57.6%)	

^a^ “other” = IgD, IgM, Biclonal, Nonsecretory

^1^ *p*-value of Mann-Whitney U test for continuous variables, Fisher’s exact test for categorical variables

Patients who would be otherwise not have been eligible for the TOURMALINE-MM1 clinical trial (due to comorbidities, performance status, refractority to any of previous regimens and number of previous regimens) were included in this analysis.

Patients received standard dosing of IRD: ixazomib 4 mg on days 1, 8 and 15, lenalidomide 25 mg on days 1 through 21 and dexamethasone 20–40 mg on days 1, 8, 15 and 22 in 28-day cycles. Reduction of any of the drugs was allowed at the discretion of the physician. All patients were required to use thromboprophylaxis as per institutional guidelines. In most centers we used antiviral prophylaxis with acyclovir 200–400 mg daily due to possible reactivation of varicella zoster virus by a proteasome inhibitor [11, 12].

### Assessments and data acquisition

The patients were followed in routine monitoring visits (usually monthly for the drug supply). The assessment of therapeutic response was performed every cycle based on the International Myeloma Working Group (IMWG) response criteria, incorporating an additional category of minimal response [13–15].

All the patients provided written, informed consent prior to participation in the program, and the study was performed under the control of the state regulatory office and local ethical committees. The data of each patient were blinded and recorded under a unique code into the Registry of Monoclonal Gammopathies (RMG), a large multicenter database collecting data of MM patients within the Central Europe [16]. All the data (including therapeutic responses as recorded by each site) are being regularly monitored with allowed error rate of <3.0%.

### Statistical analysis

The study analysis was prospectivaly defined by the statistical analysis plan. Data were described by absolute and relative frequencies of categorical variables and median (min–max) for quantitative variables. Statistical significance of differences among subgroups was assessed using the Fisher’s exact test for categorical variables and Mann-Whitney U test for continuous variables.

Survival analysis for different endpoints was conducted using the Kaplan-Meier method complemented by the 95% Greenwood confidence interval for estimates of probability survival. Statistical significance of differences in survival among subgroups was assessed using the log-rank test.

Univariable Cox proportional hazards models were used to evaluate the effect of treatment regimen on the survival measures in subgroups. Statistical significance of hazard ratios (HR) was assessed by mean of Wald test.

Multivariable Cox proportional hazards model was used to assess the significance of the treatment regimen as a predictive factor of survival independent of uneven variables (Supplementary Tables 1, 2 and 3).

All statistical tests were performed at a significance level of α = 0.05 (all tests two-sided).

Analysis was performed in SPSS software (IBM Corp. Released 2016. IBM SPSS Statistics for Windows, Version 24.0.0.1 Armonk, NY: IBM Corp.) and software R version 3.4.2 (www.r-project.org).

## Results

### Patients and previous therapy

The baseline characteristics of both the IRD and RD cohorts are summarized in Table 1. The cohorts were overall well balanced, with some statistically significant differences. The median age of the IRD cohort was slightly younger than the RD cohort (66.0 vs 68.0 years, *p* = 0.002). On the other hand, more extramedullary myeloma (EM) was recorded in the IRD cohort (14.2% vs 6.7%, *p* = 0.034). Finally, a higher proportion of patients in the IRD cohort had been pretreated with autologous stem cell transplant (ASCT) (62.0% vs 43.3%, *p* < 0.001) and proteasome inhibitor (96.9% vs 91.2%, *p* = 0.047). All other variables were evenly distributed with no significant differences between the cohorts. We tested the impact of selected variables on response rates and survival measures in a univariable analysis in order to define whether any of them had an efect on the treatment outcomes. Significant results were found in the case of “disease status” (relapsed/refractory/primary refractory/relapsed and refractory) for all, PFS, OS and ORR. In the case of age and extramedullary disease, there was a significant result for OS only. We therefore performed a multivariable and paired analysis in order to adjust for the significantly different baseline characteristics. The multivariable analysis showed no impact of uneven baseline variables on ORR, PFS and OS.

Altogether, 15 patients (5 in the IRD arm and 10 in RD arm) proceeded to ASCT and discontinued induction therapy after 3–6 cycles of lenalidomide-based induction. In order to eliminate possible bias from transplant, results were calculated for both the total cohort as well as the cohort of patients without subsequent ASCT.

The median number of previous lines of therapy was 1, with similar percentages of patients being treated after ≥4 prior lines in both cohorts (10.2% vs 7.8%). Most patients had received prior bortezomib (94.5% vs 89.9%), followed by thalidomide (43.3% vs 48.4%) and lenalidomide (17.3% vs 15.2%). Despite this being an exclusion criterion for the TOURMALINE-MM1 clinical trial, some patients in the IRD cohort had disease that was refractory to either PI- or IMiD-based therapy, including bortezomib (18.9%), thalidomide (7.1%), lenalidomide (5.5%), and carfilzomib (2.4%).

### Treatment

The proportion of the various starting doses of lenalidomide were similar in both cohorts: 25 mg (72.8% vs 63.3%), 15 mg (20.8% vs 17.2%), 10 mg (6.4% vs 16.7%), and 5 mg (0% vs 2.2%) the starting dose was significantly higher in the IRD cohort but the multivariable analysis showed no impact on the final results; 44.8% of patients underwent dose reduction in the IRD cohort compared to 37.8% in the RD cohort. The starting dose of ixazomib in IRD cohort was 4 mg in 93.6% of patients and 3 mg in 6.5% of patients; a total of 17.7% of patients underwent dose reduction for ixazomib. At the time of analysis, treatment was still ongoing in 40.2% of patients in the IRD cohort and in 34.1% of patients in the RD cohort. The most common reason for treatment withdrawal was disease progression (29.9% vs 21.2%), followed by death (5.5% vs 11.1%) and insufficient response (3.9% vs 6.0%).

### Response assessment

The registry data cut-off for this analysis was June 2019. Median follow-up was slightly longer in the IRD cohort (20.8 vs 15.5 months). The overall response rate (ORR) was 73.0% in the IRD cohort vs 66.2% in the RD cohort. Maximal treatment response rates in both cohorts are reported in Table 2: complete response (CR) (11.1% vs 8.8%), very good partial response (VGPR) (22.2% vs 13.9%), partial response (PR) (39.7% vs 43.5%) and minimal response (MR) in (8.7 vs 15.7%). Although ORR was not statistically different between cohorts, patients in the IRD cohort reached significantly more ≥VGPR responses than in the RD cohort (33.3% vs 22.7%, *p* = 0.042).

**Table 2:** Maximal treatment response

	IRD (***N*** = 127)	RD (***N*** = 217)	***p***^**1**^
**Maximal treatment response**, n (%)	*n* = 126	*n* = 216	
sCR	2 (1.6%)	–	0.095
CR	12 (9.5%)	19 (8.8%)	
VGPR	28 (22.2%)	30 (13.9%)	
PR	50 (39.7%)	94 (43.5%)	
MR	11 (8.7%)	34 (15.7%)	
SD	13 (10.3%)	17 (7.9%)	
PD	10 (7.9%)	22 (10.2%)	
VGPR+ ^a^	42 (33.3%)	49 (22.7%)	**0.042**
ORR^b^	92 (73.0%)	143 (66.2%)	0.227
CBR^c^	103 (81.7%)	177 (81.9%)	1.000

^a^ VGPR+ − patients reaching at least very good partial response

^b^ ORR – Overall Response Rate (PR or better)

^c^ CBR – Clinical Benefit Rate (MR or better)

^1^ *p*-value of Fisher’s exact test

**Fig. 1:**
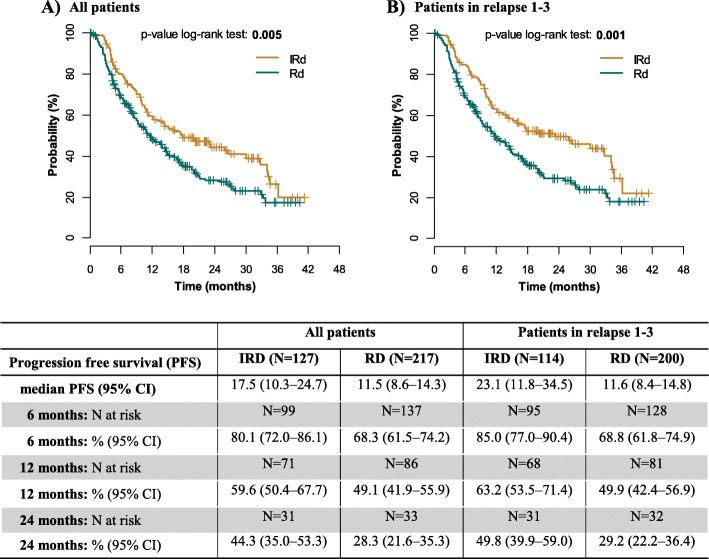
Progression free survival from treatment initiation

Median progression free survival (PFS) was significantly improved in the IRD cohort (17.5 vs 11.5 months, *p* = 0.005); in patients with 1–3 prior relapses median PFS was improved further (23.1 vs 11.6 months *p* = 0.001), as shown in Fig. 1. The hazard ratio for disease progression or death for patients in the IRD cohort compared to RD was 0.67 (95% CI 0.51–0.89, *p* = 0.006). A higher percentage of patients in the IRD cohort were progression-free at 6 months (80.1% vs 68.3%), 12 months (59.6% vs 49.1%) and 24 months (44.3% vs 28.3%).

**Fig. 2:**
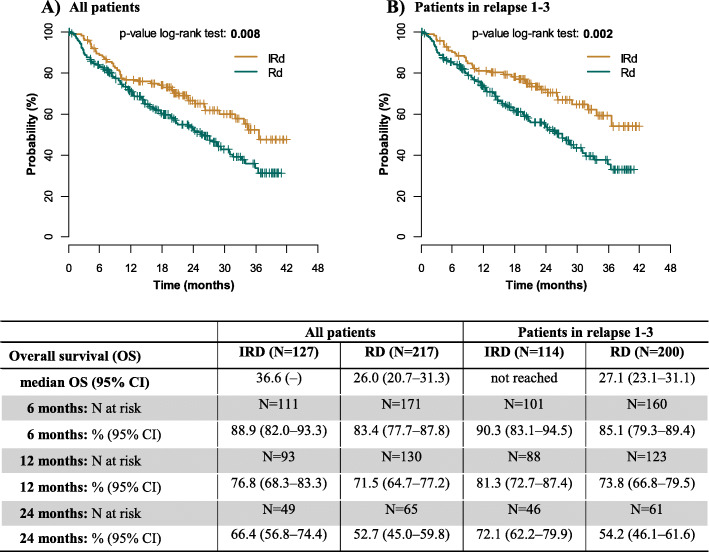
Overall survival from treatment initiation

Median OS was significantly improved in the IRD cohort (36.6 months vs 26.0 months, *p* = 0.008); in patients with 1–3 prior relapses median OS was not yet reached for the IRD cohort (NR vs 27.1 months, *p* = 0.002). A higher percentage of patients in the IRD cohort were still alive at 6 months (88.9% vs 83.4%), 12 months (76.8% vs 71.5%), and 24 months (66.4% vs 52.7%), as shown in Fig. 2.

In the analysis conducted for all patients who did not undergo subsequent ASCT (*n* = 329), response rates in both the IRD cohort (*n* = 122) and RD cohort (*n* = 207) were not different, nor did they significantly affect PFS or OS. Adjusted response rates were as follows: CR (10.8% vs 7.8%), VGPR (23.1% vs 13.6%), PR (38.8% vs 43.7%) and MR (9.1% vs 16.0%). The significant difference between cohorts for patients who reached ≥VGPR was maintained (33.9% vs 21.4%, *p* = 0.018). Among the 5 patients who were treated with IRD induction followed by ASCT, one reached CR, three reached PR and one progressed on treatment. Among the 10 patients who were treated with RD induction followed by ASCT, three reached CR, two reached VGPR, four reached PR and one reached MR.

**Fig. 3:**
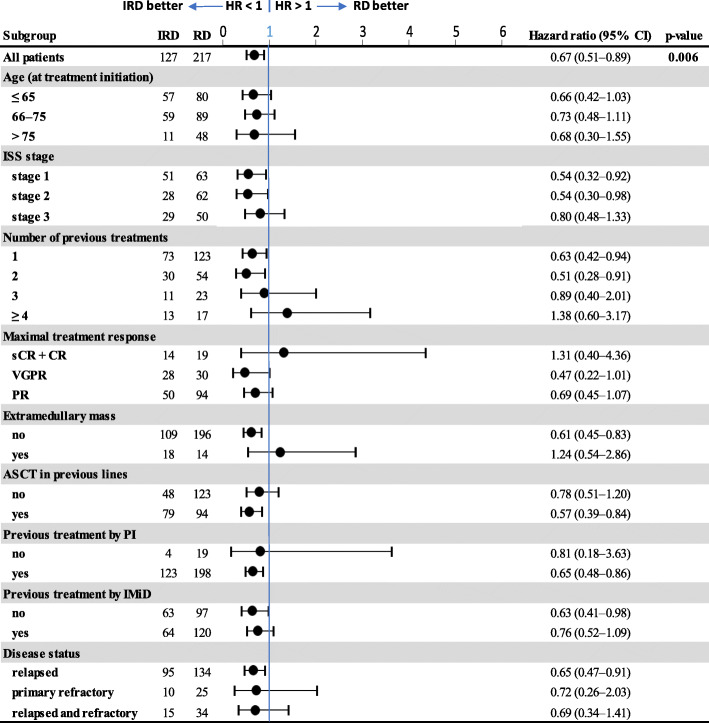
Subgroup analysis – association of IRD and RD with PFS

**Fig. 4:**
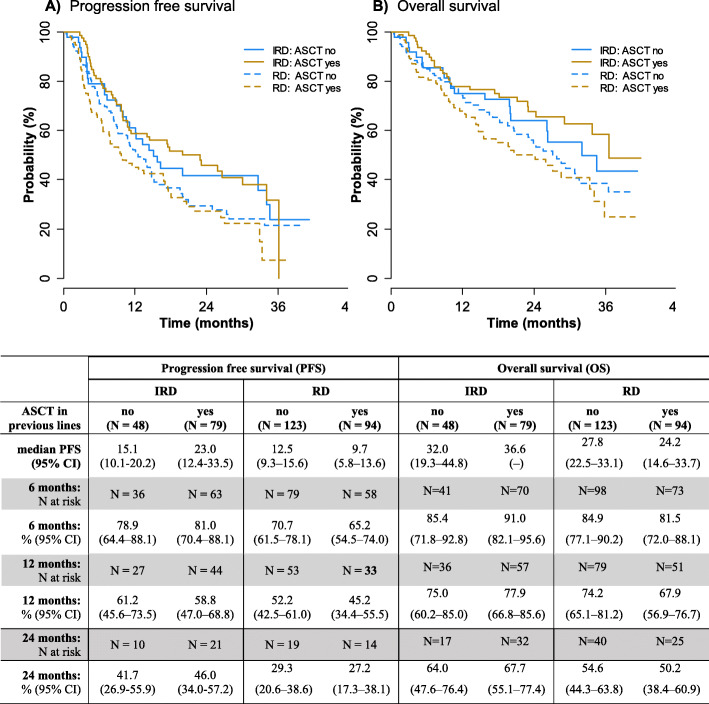
Progression free survival (**a**) and overall survival (**b**) by previous autologous stem cell transplantation (ASCT)

Patients in the the majority of pre-specified subgroups (e.g., age, ISS stage, maximal treatment response, and pretreatment) also experienced longer PFS if they were in the IRD cohort, as shown in Fig. 3. Analysis of the number of prior relapses also showed that the IRD cohort experienced longer median PFS up to the third relapse: 1st relapse (32.7 vs 14.8 months, HR 0.63 [0.42–0.94]), 2nd relapse (23.1 vs 9.3 months, HR 0.51 [0.28–0.91]), 3rd relapse (9.7 vs 9.0 months, HR 0.89 [0.40–2.01]), and ≥ 4th relapse (5.0 vs 9.9 months, HR 1.38 [0.60–3.17]). In addition, patients in the IRD cohort who had prior ASCT experienced longer median PFS than patients without previous transplant (23.0 vs 15.1 months), as shown in Figs. 3 and 4.

**Fig. 5:**
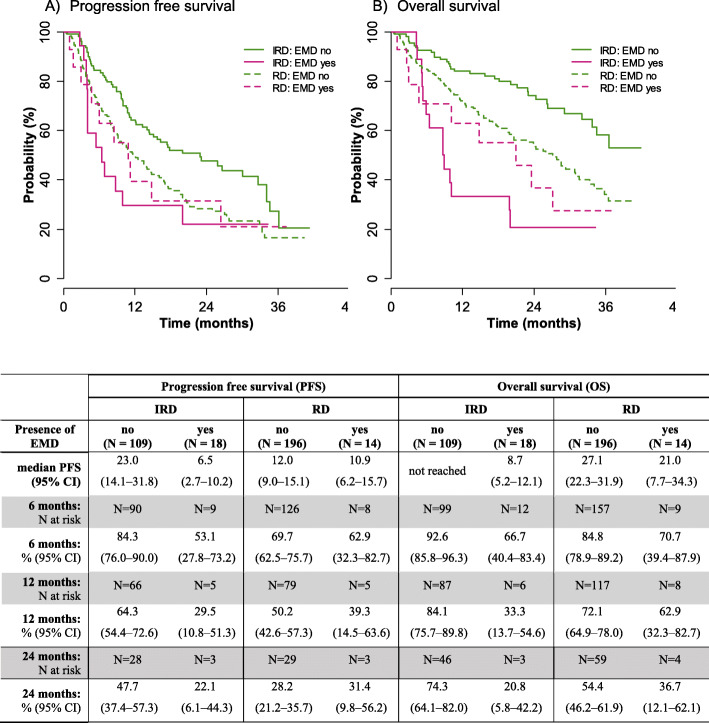
Progression free survival (**a**) and overall survival (**b**) in patients with or without extramedullary plasmocytoma (EMD) treated by IRD or RD regimen

Notably, patients in the IRD cohort with EM disease did not benefit from treatment and their median PFS was similar to patients in the RD cohort (6.5 vs 10.9 months, HR 1.24 [0.54–2.86]), as shown in Figs. 3 and 5. Two additional patient subgroups in the IRD cohort experienced at interim analysis shorter median PFS, including those with previous IMiD treatment (11.3 months) and with refractory disease in the previous line of treatment (9.7 months); however, the IRD cohort reported better hazard ratios for median PFS in both subgroups, (HR 0.76 [0.52–1.09] and 0.69 [0.34–1.41] respectively), as shown in Fig. 3.

In both cohorts, median PFS decreased with advanced ISS stage: stage 1 (32.7 vs 17.1 months, HR 0.54 [0.32–0.92]), stage 2 (25.8 vs 9.7 months, HR 0.54 [0.30–0.98]), and stage 3 (9.9 vs 6.4 months, HR 0.80 [0.48–1.33]). Although clinical outcomes were generally more favorable in younger patients, the IRD cohort reported longer median PFS regardless of age: ≤65 years (23.0 vs 10.8 months, HR 0.66 [0.42–1.03]), 66–75 years (17.8 vs 11.5 months, HR 0.73 [0.48–1.11]), and >75 years (11.1 vs 11.7 months, HR 0.68 [0.30–1.55]).

### Cytogenetics

Results from at least one cytogenetic assessment was available for 69% (87/127) of patients in the IRD cohort and 49% (106/217) of patients in the RD cohort. Cytogenetic data recorded in the registry were acquired at the time of MM diagnosis (± 6 months), as shown in Table 3. In the IRD cohort, 12.1% (8/66) of assessed patients were positive for del(17p13), 10.2% (6/59) of asssessed patients were positive for t(4;14), and 5.8% (3/52) of assessed patients were positive for t(14;16). In the RD cohort, 13.2% (12/91) of assessed patients were positive for del(17p13), 7.0% (6/86) of assessed patients were positive for t(4;14), and 4.3% (3/69) of assessed patients were positive for t(14;16). There were no significant differences in the presence of high-risk cytogenetic features between the cohorts. High-risk features as defined by the presence at least one cytogenetic abnormality including t(4;14), t(14;16), or del(17p13) were present in 11.8% (15/127) patients in the IRD cohort and 8.8% (19/217) patients in the RD cohort. Among those patients in the IRD cohort whose disease was considered high-risk, one patient reached VGPR, six reached PR, four reached MR, two had stable disease (SD) and two progressed (PD). Among those patients in the RD cohort whose disease was considere high-risk, one patient reached VGPR, seven reached PR, five reached MR, three maintained SD and three had PD.

**Table 3:** Cytogenetics

Cytogenetics^a^	IRD (***N*** = 127)	RD (***N*** = 217)	***p***^1^
**t(4;14)**, n (%)	*n* = 59	*n* = 86	
Negative	53 (89.8%)	80 (93.0%)	0.548
Positive	6 (10.2%)	6 (7.0%)	
**t(14;16)**, n (%)	*n* = 52	*n* = 69	
Negative	49 (94.2%)	66 (95.7%)	1.000
Positive	3 (5.8%)	3 (4.3%)	
**del(17p13)**, n (%)	*n* = 66	*n* = 91	
Negative	58 (87.9%)	79 (86.8%)	1.000
Positive	8 (12.1%)	12 (13.2%)	
**Cytogenetics - risk groups**^**b**^, n (%)	*n* = 54	*n* = 77	
Standard risk	39 (72.2%)	58 (75.3%)	0.691
High risk	15 (27.8%)	19 (24.7%)	

^a^ Only cytogenetics evaluated at diagnosis before treatment initiation included. Cytogenetics assessed in 69% of IRD patients (87/127) and 49% of RD patients (106/217) but most patients did not have all required parameters assessed. The table contains only the three major risk aberrations

^b^ “standard risk” = negative t(4;14) and t(14;16) and del(17p13); “high risk” = positive at least one of these aberrations

^1^ *p*-value of Fisher’s exact test for categorical variables

### Adverse events

Adverse events were graded according to the National Cancer Institute’s Common Terminology Criteria for Adverse Events (CTCAE), version 4.0. The frequency of adverse events (AEs) associated with IRD and RD was consistent with published clinical trial experience [1]. The majority of grade ≥ 3 AEs included hematological toxicity, and there was no statistically significant difference between the occurence of cytopenia between the cohorts, as seen in Table 4. Grade ≥ 3 anemia was reported in 12.0% vs 25.6%, neutropenia in 28.4% vs 23.1% and thrombocytopenia in 21.3% vs 22.6% of patients in the cohorts, respectively. The IRD cohort reported significantly more episodes of grade 1 and 2 infection (50.0% vs 29.1%, *p* = 0.005) but the rates of grade ≥ 3 infection was similar in both cohorts (21.4% vs 22.5%). Similarly, the IRD cohort reported slightly more cases of peripheral neuropathy (PN), but grade ≥ 3 neuropathy was recorded for only three patients and most PN cases were associated with previous pretreatment with bortezomib or thalidomide. Grade 1 PN in both cohorts was present in 36.0% vs 32.8%, grade 2 in 16.0% vs 7.8%, grade 3 in 4.0% vs 0%, the difference between the cohorts was small but significant (*p* = 0.011). We looked thoroughly at “regimen specific toxicities” (i.e., non-hematological toxicities that were most often reported by other trials in association with ixazomib treatment): fatigue, nausea and vomiting, diarrhea and rash. Among these, only diarrhea was significantly more frequent in the IRD cohort but only 1 patient had grade ≥ 3 diarrhea. The reason for treatment discontinuation due to toxicity was in 3.1% in the IRD cohort (4/127) and 4.1% in the RD cohort (9/217).

**Table 4:** Toxicities

Toxicity^**a**^, n (%)	Grade of toxicity			***p***^1^		
	G1	G2	≥ G3	G0 vs G1–2 vs G3–5	G1–2 vs other	G3–5 vs other
**Anemia**						
IRD (*n* = 75)	32 (42.7%)	23 (30.7%)	9 (12.0%)	0.065	0.093	**0.021**
RD (*n* = 129)	43 (33.3%)	36 (27.9%)	33 (25.6%)			
**Neutropenia**						
IRD (*n* = 74)	22 (29.7%)	13 (17.6%)	21 (28.4%)	0.701	0.771	0.406
RD (*n* = 130)	48 (36.9%)	17 (13.1%)	30 (23.1%)			
**Thrombocytopenia**						
IRD (*n* = 75)	23 (30.7%)	9 (12.0%)	16 (21.3%)	0.744	0.661	0.863
RD (*n* = 128)	46 (35.9%)	14 (10.9%)	29 (22.6%)			
**Thrombosis/** **Thrombus/Embolism**						
IRD (*n* = 64)	3 (4.7%)	–	3 (4.7%)	0.460	1.000	0.349
RD (*n* = 116)	6 (5.2%)	1 (0.9%)	2 (1.7%)			
**Fatigue**						
IRD (*n* = 73)	18 (24.7%)	29 (39.7%)	5 (6.8%)	0.744	0.547	0.607
RD (*n* = 127)	28 (22.0%)	47 (37.0%)	12 (9.4%)			
**Infection**						
IRD (*n* = 70)	9 (12.9%)	26 (37.1%)	15 (21.4%)	**0.008**	**0.005**	1.000
RD (*n* = 124)	11 (8.9%)	25 (20.2%)	28 (22.5%)			
**Neuropathy**^**b**^						
IRD (*n* = 75)	27 (36.0%)	12 (16.0%)	3 (4.0%)	**0.011**	0.144	**0.049**
RD (*n* = 128)	42 (32.8%)	10 (7.8%)	–			
**Nausea, vomiting**						
IRD (*n* = 67)	21 (31.3%)	11 (16.4%)	–	0.062	**0.041**	0.535
RD (*n* = 119)	25 (21.2%)	13 (11.0%)	2 (1.7%)			
**Anorexia**						
IRD (*n* = 67)	19 (28.4%)	3 (4.5%)	–	0.400	0.398	0.537
RD (*n* = 119)	17 (14.3%)	14 (11.8%)	2 (1.7%)			
**Diarrhea**						
IRD (*n* = 69)	14 (20.3%)	9 (13.0%)	1 (1.4%)	**0.026**	0.052	0.369
RD (*n* = 118)	12 (10.2%)	11 (9.3%)	–			
**Constipation**						
IRD (*n* = 65)	7 (10.8%)	1 (1.5%)	–	1.000	1.000	–
RD (*n* = 115)	8 (7.0%)	6 (5.2%)	–			
**Exanthema/rash**						
IRD (*n* = 4)	–	3 (75.0%)	1 (25.0%)	1.000	1.000	1.000
RD (*n* = 2)	–	2 (100.0%)	–			
**Other (max. grade)**						
IRD (*n* = 48)	5 (10.4%)	34 (70.8%)	9 (18.8%)	0.185	0.185	0.185
RD (*n* = 60)	9 (15.0%)	32 (53.3%)	19 (31.7%)			

^a^ Evaluated only for patients with terminated treatment IRD or RD. Including patients (*N* = 1 for IRD and *N* = 4 for RD) who were excluded from other analyses due to switch associated with toxicity

^b^Includes both previous and newly acquired neuropathy

^1^ *p*-value of Fisher’s exact test

## Discussion

A major disadvantage of most current treatment options for patients with MM (apart from possible adverse effects) is the need for parenteral application which requires frequent hospital visits. The TOURMALINE-MM1 clinical trial introduced a fully-oral combination of a proteasome inhibitor (ixazomib), immunomodulatory drug (lenalidomide) and a steroid (dexamethasone) [1]. The IRD regimen provided a significant benefit in response rates and median PFS, as well as demonstrated a feasible toxicity profile and no significant decrease in quality of life; it was later approved by the FDA and EMA for the treatment of patients with MM who have relapsed after at least one prior line of therapy [1].

This current analysis lends support to the therapeutic value of the IRD regimen within a real-world setting, and is consistent with other recent studies [17–20]. Notably, despite patients in this study being unselected according to typical clinical trial inclusion/exclusion criteria, these outcomes are consistent with those published from the TOURMALINE-MM1 trial. Moreover, this analysis of real-world patient experience showed first time that improvement of median PFS also was associated with significantly improved OS in the IRD cohort, which is a novel discovery in an unselected patient population.

In this analysis, patients in most prespecified subgroups reported an improved PFS in the IRD cohort when compared to patients in the RD cohort; unlike the TOURMALINE-MM1 trial, we did not observe worse outcomes of the IRD regimen in patients undergoing ASCT [7]. However, this analysis showed that patients with higher pretreatment received less benefit from IRD than patients treated in early relapses. In addition, patients with the presence of extramedullary plasmocytoma had significantly worse outcomes that were comparable in the IRD arm than in the RD cohort (median PFS 5.5 vs 11.2 months). This analysis also demonstrated less favorable outcomes for patients with refractory disease and those with prior IMiD exposure, two populations which were excluded by the TOURMALINE-MM1.

The toxicities reported by patients in the IRD cohort were largely hematological, but most did not significantly differ from the RD cohort. Patients in the IRD cohort had a significantly higher number of infections but the difference in grade ≥ 3 infections was negligible. The TOURMALINE-MM1 trial excluded patients with peripheral neuropathy grade 1 with pain or grade 2 and higher. In our study, there were more patients with preexisting grade ≥ 2 neuropathy in the IRD compared to the RD cohorts (14.9% vs 8.5%). After treatment, there was a slight but significant increase in grade ≥ 2 neuropathy in the IRD compared to the RD cohorts (20% vs 8%). However, similarly to the TOURMALINE-MM1 trial and recent real world observations, no patient quit the treatment due to PN, showing that the drug combination is feasible even in patients with pre-existing peripheral neuropathy [1, 17, 20]. In addition, this analysis showed that IRD did not increase the rate of treatment termination due to AEs when compared to RD (3.1% vs 4.1%), thus supporting the conclusion that administration and management of IRD in the real-world setting is associated with very low treatment withdrawal due to toxicities.

The current standard of care in patients with RRMM include several drug combinations, among which lenalidomide-based triplets have shown the most beneficial results [21]. At the moment, there are five FDA-approved lenalidomide-based triplet combinations which combine lenalidomide/dexamethasone to novel agents: bortezomib (RVD), carfilzomib (KRD), elotuzumab (ERD), ixazomib (IRD), and most recently daratumumab (DRD). Although their respective clinical trials are not directly comparable, the response rates as well as survival measures are quite similar and all with better results than their comparator RD [1, 22–28]. However, the choice of individual treatment modality cannot be based merely on direct regimen comparisons from randomized clinical trials, and the real world setting requires additional considerations, including cost, availability, and patient preference. Especially recent pandemy of SARS-CoV-2 showed that the all-oral IRD regimen offers an efficient and tolerable treatment modality with low frequency of hospital visits.

The nature of this analysis as well as the lack of randomization or placebo control are obvious limitations. Nevertheless, the availability of the NPP with uniform national guidelines and treatment schedules allowed for salient comparison between the two treatment cohorts. There were no major statistically significant differences in most aspects of the demographics, disease type and stage, and previous and concommitant treatment schedules. Possible sources of bias might be due to the differences in the patients’ age, pretreatment by ASCT and dose of lenalidomide. Patients in the IRD cohort were slightly but significantly younger which might have contributed to the outcomes as well as the finding that better hazard ratios were seen in patients <75 years of age. The difference in ASCT pretreatment may be attributable to the lower age in the IRD cohort, too. And, the induction dose of lenalidomide was also higher in the IRD cohort (25 mg starting dose was in 72.8% vs 63.3%, respectively), contributing to possible bias. Both univariable and multivariable analysis were therefore performed to assess the possible impact of any uneven variables in baseline differences, which confirmed that these did not have significant effect on the final results.

## Conclusions

In summary, this real-world comparison is consistent with the conclusions of the TOURMALINE-MM1 trial, with some notable exceptions. Contrary to the clinical trial experience, patients in this study reported better outcomes associated with IRD in the first relapse setting compared to later relapses, non-inferior outcomes from IRD in patients who received prior ASCT, and poorer outcomes from IRD treatment in patients with extramedullary disease. The present analysis shows that for patients with RRMM treated in routine clinical practice, IRD is well tolerated and was associated with better PFS and OS when compared to RD.

## Supplementary Information

**Additional file 1: Supplementary Table 1a.** Association of PFS with selected variables. **Supplementary Table 1b** Association of PFS with selected variables in multivariable analysis – Paired analysis.

**Additional file 2: Supplementary Table 2a.** Association of OS with selected variables. **Supplementary Table 2b** Association of OS with selected variables in multivariable analysis – Paired analysis.

**Additional file 3: Supplementary Table 3a.** Association of ORR with selected variables. **Supplementary Table 3b** Association of ORR with selected variables in multivariable analysis – Paired analysis.

## Data Availability

The data of each patient were blinded and recorded under a unique code into the Registry of Monoclonal Gammopathies (RMG), a large multicenter database collecting data of MM patients within the Central Europe. The datasets generated and/or analysed during the current study are not publicly available as local restrictions apply to the availability but are available from the corresponding author on reasonable request and with permission of all cooperating centres.

## Abbreviations

AEs: Adverse events
ASCT: Autologous stem cell transplant
CR: Complete response
CTCAE: The National Cancer Institute’s Common Terminology Criteria for Adverse Events
DRD: Daratumumab, lenalidomide, dexamethasone
ECOG: Eastern Cooperative Group performance status
EM: Extramedullary myeloma
EMA: European Medicines Agency
ERD: Elotuzumab, lenalidomide, dexamethasone
FDA: Food and Drug Administration
HR: Hazard ratio
IMiD: Immunomodulatory drug
IMWG: International Myeloma Working Group
IRD: Ixazomib, lenalidomide, dexamethasone
ISS: International Staging System
KRD: Carfilzomib, lenalidomide, dexamethasone
MM: Multiple myeloma
MR: Minimal response
NPP: Named Patient Programme
NR: Not reached
ORR: Overall response rate
OS: Overall survival
PD: Progressive disease
PFS: Progression free survival
PI: Proteasome inhibitor
PN: Peripheral neuropathy
PR: Partial response
RD: Lenalidomide, dexamethasone
RMG: The Czech Registry of Monoclonal Gammopathies
RRMM: Relapsed and refractory multiple myeloma
RVD: Lenalidomide, bortezomib, dexamethasone
SD: Stable disease
VGPR: Very good partial presponse

## References

[1] Moreau P, Masszi T, Grzasko N (2016). Oral ixazomib, lenalidomide, and dexamethasone for multiple myeloma. N Engl J Med.

[2] Leleu X, Masszi T, Bahlis NJ, et al. Patient-reported health-related quality of life from the phase III TOURMALINE-MM1 study of ixazomib-lenalidomide-dexamethasone versus placebo-lenalidomide-dexamethasone in relapsed/refractory multiple myeloma. Am J Hematol. 2018. 10.1002/ajh.25134. 10.1002/ajh.25134 29726031

[3] Kumar SK, Berdeja JG, Niesvizky R (2014). Safety and tolerability of ixazomib, an oral proteasome inhibitor, in combination with lenalidomide and dexamethasone in patients with previously untreated multiple myeloma: an open-label phase 1/2 study. Lancet Oncol.

[4] Kumar SK, LaPlant BR, Reeder CB (2016). Randomized phase 2 trial of ixazomib and dexamethasone in relapsed multiple myeloma not refractory to bortezomib. Blood..

[5] Kumar S, Moreau P, Hari P (2017). Management of adverse events associated with ixazomib plus lenalidomide/dexamethasone in relapsed/refractory multiple myeloma. Br J Haematol.

[6] Avet-Loiseau H, Bahlis NJ, Chng WJ (2017). Ixazomib significantly prolongs progression-free survival in high-risk relapsed/refractory myeloma patients. Blood..

[7] Mateos MV, Masszi T, Grzasko N (2017). Impact of prior therapy on the efficacy and safety of oral ixazomib-lenalidomide-dexamethasone vs. placebo-lenalidomide-dexamethasone in patients with relapsed/refractory multiple myeloma in TOURMALINE-MM1. Haematologica..

[8] Millennium Pharmaceuticals Inc. NINLARO (ixazomib) capsules for oral use. United States Prescribing Information. 2016. https://www.ninlaro.com/prescribing-information.pdf. Accessed 12 Feb 2019.

[9] Richardson PG, San Miguel JF, Moreau P (2018). Interpreting clinical trial data in multiple myeloma: translating findings to the real-world setting. Blood Cancer J.

[10] Costa LJ, Hari PN, Kumar SK (2016). Differences between unselected patients and participants in multiple myeloma clinical trials in US: a threat to external validity. Leuk Lymphoma.

[11] Minarik J, Pika T, Bacovsky J, Langova K, Scudla V (2012). Low-dose acyclovir prophylaxis for bortezomib-induced herpes zoster in multiple myeloma patients. Br J Haematol.

[12] Pour L, Adam Z, Buresova L (2009). Varicella-zoster virus prophylaxis with low-dose acyclovir in patients with multiple myeloma treated with bortezomib. Clin Lymphoma Myeloma.

[13] Blade J, Samson D, Reece D (1998). Criteria for evaluating disease response and progression in patients with multiple myeloma treated by high-dose therapy and haemopoietic stem cell transplantation. Myeloma subcommittee of the EBMT. European Group for Blood and Marrow Transplant. Br J Haematol.

[14] Rajkumar SV, Harousseau JL, Durie B (2011). Consensus recommendations for the uniform reporting of clinical trials: report of the international myeloma workshop consensus panel 1. Blood..

[15] Durie BG, Harousseau JL, Miguel JS (2006). International uniform response criteria for multiple myeloma. Leukemia..

[16] Radocha J, Pour L, Spicka I (2015). Registry of monoclonal Gammopathies (RMG) in the Czech Republic. Blood..

[17] Terpos E, Maouche N, Minarik J (2017). “Real world” data on the efficacy and safety of ixazomib in combination with lenalidomide and dexamethasone in relapsed/refractory multiple myeloma: a combined study from the Greek, Czech and UK Databases. Blood.

[18] Varga G, Nagy Z, Demeter J (2019). Real world efficacy and safety results of ixazomib lenalidomide and dexamethasone combination in relapsed/refractory multiple myeloma: data collected from the Hungarian ixazomib named patient program. Pathol Oncol Res.

[19] Hajek R, Terpos E, Lee HC (2018). Ixazomib plus lenalidomide-dexamethasone (IRd) in relapsed/refractory multiple myeloma (MM) patients (Pts) - effectiveness in routine clinical practice is similar to the efficacy in the phase 3 tourmaline-MM1 trial: a pooled analysis from the insight MM observational study and the Czech registry of monoclonal gammopathies (RMG). Blood.

[20] Minarik J, Krhovska P, Jelinek T (2018). Treatment of relapsed and refractory multiple myeloma with fully oral triplet IRD (ixazomib, lenalidomide and dexamethasone) is safe and with significant therapeutic outcomes. Blood.

[21] van Beurden-Tan CHY, Franken MG, Blommestein HM, Uyl-de Groot CA, Sonneveld P (2017). Systematic literature review and network meta-analysis of treatment outcomes in relapsed and/or refractory multiple myeloma. J Clin Oncol.

[22] Richardson PG, Xie W, Jagannath S (2014). A phase 2 trial of lenalidomide, bortezomib, and dexamethasone in patients with relapsed and relapsed/refractory myeloma. Blood..

[23] Stewart AK, Rajkumar SV, Dimopoulos MA (2015). Carfilzomib, lenalidomide, and dexamethasone for relapsed multiple myeloma. N Engl J Med.

[24] Siegel DS, Dimopoulos MA, Ludwig H (2018). Improvement in overall survival with carfilzomib, lenalidomide, and dexamethasone in patients with relapsed or refractory multiple myeloma. J Clin Oncol.

[25] Lonial S, Dimopoulos M, Palumbo A (2015). Elotuzumab therapy for relapsed or refractory multiple myeloma. N Engl J Med.

[26] Dimopoulos MA, San-Miguel J, Belch A (2018). Daratumumab plus lenalidomide and dexamethasone versus lenalidomide and dexamethasone in relapsed or refractory multiple myeloma: updated analysis of POLLUX. Haematologica..

[27] Dimopoulos MA, Oriol A, Nahi H (2016). Daratumumab, Lenalidomide, and dexamethasone for multiple myeloma. N Engl J Med.

[28] Bahlis N, Dimopoulos MA, White DJ (2018). Three-year follow up of the phase 3 pollux study of daratumumab plus lenalidomide and dexamethasone (D-Rd) versus lenalidomide and dexamethasone (Rd) alone in relapsed or refractory multiple myeloma (RRMM). Blood.

