# Supplementary material for: Survival benefit of ixazomib, lenalidomide and dexamethasone (IRD) over lenalidomide and dexamethasone (Rd) in relapsed and refractory multiple myeloma patients in routine clinical practice
Source: BMC Cancer. 2021 Jan 15;21:73. doi: 10.1186/s12885-020-07732-1 (PMC7810195; doi:10.1186/s12885-020-07732-1)
Supplement: Supplementary file 1 — Additional file 1: Supplementary Table 1a. Association of PFS with selected variables. Supplementary Table 1b Association of PFS with selected variables in multivariable analysis – Paired analysis. [file 12885_2020_7732_MOESM1_ESM.zip › Supplementary 1aR3.docx]

**Supplementary table 1a** Association of PFS with selected variables

|  |  | **Cox proportional hazard model** | | | | | | |  |  |
| --- | --- | --- | --- | --- | --- | --- | --- | --- | --- | --- |
|  |  | **Univariable** | | |  | **Multivariable** | | |  |  |
|  |  | **N** | **Hazard ratio (95% CI)** | **p-value** |  | **N** | **Hazard ratio (95% CI)** | **p-value** |  |  |
| **Regimen** |  |  |  |  |  |  |  |  |  |  |
| IRD |  | 127 | reference | – |  | 118 | reference | – |  |  |
| RD |  | 217 | 0.67 (0.51–0.89) | **0,006** |  | 158 | 0.72 (0.52–0.99) | **0,042** |  |  |
| **Age (at treatment initiation)** |  |  |  |  |  |  |  |  |  |  |
| **≤ 65** |  | 137 | reference | – |  | 105 | reference | – |  |  |
| **66–75** |  | 148 | 0.92 (0.68–1.24) | 0,583 |  | 122 | 0.90 (0.62–1.30) | 0,569 |  |  |
| **> 75** |  | 59 | 1.42 (0.97–2.07) | 0,069 |  | 49 | 1.45 (0.84–2.50) | 0,182 |  |  |
| **Extramedullary mass** |  |  |  |  |  |  |  |  |  |  |
| **no** |  | 305 | reference | – |  | 248 | reference | – |  |  |
| **yes** |  | 32 | 1.42 (0.92–2.19) | 0,114 |  | 28 | 1.34 (0.84–2.13) | 0,226 |  |  |
| **ASCT in previous lines** |  |  |  |  |  |  |  |  |  |  |
| **no** |  | 171 | reference | – |  | 135 | reference | – |  |  |
| **yes** |  | 173 | 1.00 (0.76–1.30) | 0,976 |  | 141 | 1.23 (0.82–1.85) | 0,322 |  |  |
| **Previous treatment by PI** |  |  |  |  |  |  |  |  |  |  |
| **no** |  | 23 | reference | – |  | 19 | reference | – |  |  |
| **yes** |  | 321 | 1.11 (0.65–1.91) | 0,703 |  | 257 | 0.93 (0.51–1.69) | 0,809 |  |  |
| **Disease status** |  |  |  |  |  |  |  |  |  |  |
| **relapsed** |  | 229 | reference | – |  | 203 | reference | – |  |  |
| **primary refractory** |  | 35 | 1.01 (0.62–1.65) | 0,965 |  | 30 | 1.21 (0.70–2.10) | 0,497 |  |  |
| **relapsed and refractory** |  | 49 | 2.15 (1.49–3.09) | **<0.001** |  | 43 | 2.20 (1.48–3.28) | **<0.001** |  |  |
| **Lenalidomide dose (at treatment initiation)** |  |  |  |  |  |  |  |  |  |  |
| **≤ 10** |  | 42 | reference | – |  | 39 | reference | – |  |  |
| **11–20** |  | 58 | 0.91 (0.56–1.47) | 0,691 |  | 53 | 1.31 (0.78–2.20) | 0,315 |  |  |
| **> 20** |  | 205 | 0.71 (0.47–1.07) | 0,104 |  | 184 | 0.84 (0.54–1.33) | 0,462 |  |  |
| *Results from Cox proportional hazard model* | |  |  |  |  |  |  |  |  |  |
|  |  |  |  |  |  |  |  |  |  |  |
