# Supplementary material for: Survival benefit of ixazomib, lenalidomide and dexamethasone (IRD) over lenalidomide and dexamethasone (Rd) in relapsed and refractory multiple myeloma patients in routine clinical practice
Source: BMC Cancer. 2021 Jan 15;21:73. doi: 10.1186/s12885-020-07732-1 (PMC7810195; doi:10.1186/s12885-020-07732-1)
Supplement: Supplementary file 1 — Additional file 1: Supplementary Table 1a. Association of PFS with selected variables. Supplementary Table 1b Association of PFS with selected variables in multivariable analysis – Paired analysis. [file 12885_2020_7732_MOESM1_ESM.zip › Supplementary 1bR3.docx]

**Supplementary table 1b** Association of PFS with selected variables in multivariable analysis – Paired analysis

|  |  | **Paired analysis - Regimen and selected variable** | | | | | | | | | | | | | | | | | | |
| --- | --- | --- | --- | --- | --- | --- | --- | --- | --- | --- | --- | --- | --- | --- | --- | --- | --- | --- | --- | --- |
| **Subgroup** |  | **N** | **Hazard ratio (95% CI)** | **p-value** |  | **N** | **Hazard ratio (95% CI)** | **p-value** |  | **N** | **Hazard ratio (95% CI)** | **p-value** |  | **N** | **Hazard ratio (95% CI)** | **p-value** |  | **N** | **Hazard ratio (95% CI)** | **p-value** |
| **Regimen** |  |  |  |  |  |  |  |  |  |  |  |  |  |  |  |  |  |  |  |  |
| RD |  | 127 | reference |  |  | 127 | reference |  |  | 127 | reference |  |  | 127 | reference |  |  | 120 | reference |  |
| IRD |  | 217 | 0.70 (0.52–0.93) | **0.013** |  | 210 | 0.67 (0.51–0.89) | **0.006** |  | 217 | 0.66 (0.49–0.88) | **0.004** |  | 217 | 0.66 (0.50–0.88) | **0.004** |  | 193 | 0.67 (0.50–0.90) | **0.007** |
| **Age (at treatment initiation)** |  |  |  |  |  |  |  |  |  |  |  |  |  |  |  |  |  |  |  |  |
| ≤ 65 |  | 137 | reference |  |  |  |  |  |  |  |  |  |  |  |  |  |  |  |  |  |
| 66–75 |  | 148 | 0.91 (0.68–1.23) | 0.532 |  |  |  |  |  |  |  |  |  |  |  |  |  |  |  |  |
| > 75 |  | 59 | 1.30 (0.89–1.90) | 0.180 |  |  |  |  |  |  |  |  |  |  |  |  |  |  |  |  |
| **Extramedullary mass** |  |  |  |  |  |  |  |  |  |  |  |  |  |  |  |  |  |  |  |  |
| no |  |  |  |  |  | 305 | reference |  |  |  |  |  |  |  |  |  |  |  |  |  |
| yes |  |  |  |  |  | 32 | 1.46 (0.95–2.26) | 0.086 |  |  |  |  |  |  |  |  |  |  |  |  |
| **ASCT in previous lines** |  |  |  |  |  |  |  |  |  |  |  |  |  |  |  |  |  |  |  |  |
| no |  |  |  |  |  |  |  |  |  | 171 | reference |  |  |  |  |  |  |  |  |  |
| yes |  |  |  |  |  |  |  |  |  | 173 | 1.10 (0.83–1.45) | 0.506 |  |  |  |  |  |  |  |  |
| **Previous treatment by PI** |  |  |  |  |  |  |  |  |  |  |  |  |  |  |  |  |  |  |  |  |
| no |  |  |  |  |  |  |  |  |  |  |  |  |  | 23 | reference |  |  |  |  |  |
| yes |  |  |  |  |  |  |  |  |  |  |  |  |  | 321 | 1.26 (0.73–2.18) | 0.415 |  |  |  |  |
| **Disease status** |  |  |  |  |  |  |  |  |  |  |  |  |  |  |  |  |  |  |  |  |
| relapsed |  |  |  |  |  |  |  |  |  |  |  |  |  |  |  |  |  | 229 | reference |  |
| primary refractory |  |  |  |  |  |  |  |  |  |  |  |  |  |  |  |  |  | 35 | 0.97 (0.59–1.58) | 0.886 |
| relapsed and refractory |  |  |  |  |  |  |  |  |  |  |  |  |  |  |  |  |  | 49 | 2.09 (1.45–3.02) | **< 0.001** |

*Results from Cox proportional hazard model*
