# Supplementary material for: Survival benefit of ixazomib, lenalidomide and dexamethasone (IRD) over lenalidomide and dexamethasone (Rd) in relapsed and refractory multiple myeloma patients in routine clinical practice
Source: BMC Cancer. 2021 Jan 15;21:73. doi: 10.1186/s12885-020-07732-1 (PMC7810195; doi:10.1186/s12885-020-07732-1)
Supplement: Supplementary file 2 — Additional file 2: Supplementary Table 2a. Association of OS with selected variables. Supplementary Table 2b Association of OS with selected variables in multivariable analysis – Paired analysis. [file 12885_2020_7732_MOESM2_ESM.zip › Supplementary 2aR3.docx]

**Supplementary table 2a** Association of OS with selected variables

|  |  |  | **Cox proportional hazard model** | | | | | | |
| --- | --- | --- | --- | --- | --- | --- | --- | --- | --- |
|  |  |  | **Univariable analysis** | | |  | **Multivariable analysis** | | |
|  |  |  | **N** | **Hazard ratio (95% CI)** | **p-value** |  | **N** | **Hazard ratio (95% CI)** | **p-value** |
|  | **Regimen** |  |  |  |  |  |  |  |  |
|  | IRD |  | 127 | reference | – |  | 118 | reference | – |
|  | RD |  | 217 | 0.63 (0.45–0.89) | **0,009** |  | 158 | 0.64 (0.43–0.95) | **0,028** |
|  | **Age (at treatment initiation)** |  |  |  |  |  |  |  |  |
|  | **≤ 65** |  | 137 | reference | – |  | 105 | reference | – |
|  | **66–75** |  | 148 | 1.03 (0.72–1.47) | 0,894 |  | 122 | 0.98 (0.62–1.56) | 0,942 |
|  | **> 75** |  | 59 | 1.58 (1.01–2.45) | **0,044** |  | 49 | 1.50 (0.79–2.84) | 0,218 |
|  | **Extramedullary mass** |  |  |  |  |  |  |  |  |
|  | **no** |  | 305 | reference | – |  | 248 | reference | – |
|  | **yes** |  | 32 | 2.32 (1.48–3.62) | **<0.001** |  | 28 | 2.24 (1.37–3.66) | **0,001** |
|  | **ASCT in previous lines** |  |  |  |  |  |  |  |  |
|  | **no** |  | 171 | reference | – |  | 135 | reference | – |
|  | **yes** |  | 173 | 0.96 (0.70–1.33) | 0,821 |  | 141 | 1.22 (0.74–1.99) | 0,439 |
|  | **Previous treatment by PI** |  |  |  |  |  |  |  |  |
|  | **no** |  | 23 | reference | – |  | 19 | reference | – |
|  | **yes** |  | 321 | 0.99 (0.55–1.79) | 0,978 |  | 257 | 1.12 (0.58–2.15) | 0,738 |
|  | **Disease status** |  |  |  |  |  |  |  |  |
|  | **relapsed** |  | 229 | reference | – |  | 203 | reference | – |
|  | **primary refractory** |  | 35 | 1.21 (0.70–2.09) | 0,496 |  | 30 | 1.26 (0.68–2.31) | 0,466 |
|  | **relapsed and refractory** |  | 49 | 2.67 (1.81–3.93) | **<0.001** |  | 43 | 2.54 (1.65–3.90) | **<0.001** |
|  | **Lenalidomide dose (at treatment initiation)** |  |  |  |  |  |  |  |  |
|  | **≤ 10** |  | 42 | reference | – |  | 39 | reference | – |
|  | **11–20** |  | 58 | 0.81 (0.47–1.40) | 0,445 |  | 53 | 1.23 (0.67–2.26) | 0,498 |
|  | **> 20** |  | 205 | 0.61 (0.38–0.98) | 0,040 |  | 184 | 0.79 (0.47–1.34) | 0,387 |
|  | *Results from Cox proportional hazard model* | |  |  |  |  |  |  |  |
|  |  |  |  |  |  |  |  |  |  |
