# Supplementary material for: Survival benefit of ixazomib, lenalidomide and dexamethasone (IRD) over lenalidomide and dexamethasone (Rd) in relapsed and refractory multiple myeloma patients in routine clinical practice
Source: BMC Cancer. 2021 Jan 15;21:73. doi: 10.1186/s12885-020-07732-1 (PMC7810195; doi:10.1186/s12885-020-07732-1)
Supplement: Supplementary file 2 — Additional file 2: Supplementary Table 2a. Association of OS with selected variables. Supplementary Table 2b Association of OS with selected variables in multivariable analysis – Paired analysis. [file 12885_2020_7732_MOESM2_ESM.zip › Supplementary 2bR3.docx]

**Supplementary table 2b** Association of OS with selected variables in multivariable analysis – Paired analysis

|  |  | **Paired analysis - Regimen and selected variable** | | | | | | | | | | | | | | | | | | |
| --- | --- | --- | --- | --- | --- | --- | --- | --- | --- | --- | --- | --- | --- | --- | --- | --- | --- | --- | --- | --- |
| **Subgroup** |  | **N** | **Hazard ratio (95% CI)** | **p-value** |  | **N** | **Hazard ratio (95% CI)** | **p-value** |  | **N** | **Hazard ratio (95% CI)** | **p-value** |  | **N** | **Hazard ratio (95% CI)** | **p-value** |  | **N** | **Hazard ratio (95% CI)** | **p-value** |
| **Regimen** |  |  |  |  |  |  |  |  |  |  |  |  |  |  |  |  |  |  |  |  |
| RD |  | 127 | reference |  |  | 127 | reference |  |  | 127 | reference |  |  | 127 | reference |  |  | 120 | reference |  |
| IRD |  | 217 | 0.66 (0.47–0.94) | **0.022** |  | 210 | 0.61 (0.43–0.87) | **0.006** |  | 217 | 0.62 (0.44–0.88) | **0.008** |  | 217 | 0.62 (0.44–0.88) | **0.008** |  | 193 | 0.62 (0.43–0.88) | **0.007** |
| **Age (at treatment initiation)** |  |  |  |  |  |  |  |  |  |  |  |  |  |  |  |  |  |  |  |  |
| ≤ 65 |  | 137 | reference |  |  |  |  |  |  |  |  |  |  |  |  |  |  |  |  |  |
| 66–75 |  | 148 | 1.03 (0.72–1.48) | 0.874 |  |  |  |  |  |  |  |  |  |  |  |  |  |  |  |  |
| > 75 |  | 59 | 1.43 (0.91–2.24) | 0.121 |  |  |  |  |  |  |  |  |  |  |  |  |  |  |  |  |
| **Extramedullary mass** |  |  |  |  |  |  |  |  |  |  |  |  |  |  |  |  |  |  |  |  |
| no |  |  |  |  |  | 305 | reference |  |  |  |  |  |  |  |  |  |  |  |  |  |
| yes |  |  |  |  |  | 32 | 2.42 (1.55–3.78) | **< 0.001** |  |  |  |  |  |  |  |  |  |  |  |  |
| **ASCT in previous lines** |  |  |  |  |  |  |  |  |  |  |  |  |  |  |  |  |  |  |  |  |
| no |  |  |  |  |  |  |  |  |  | 171 | reference |  |  |  |  |  |  |  |  |  |
| yes |  |  |  |  |  |  |  |  |  | 173 | 1.07 (0.77–1.48) | 0.693 |  |  |  |  |  |  |  |  |
| **Previous treatment by PI** |  |  |  |  |  |  |  |  |  |  |  |  |  |  |  |  |  |  |  |  |
| no |  |  |  |  |  |  |  |  |  |  |  |  |  | 23 | reference |  |  |  |  |  |
| yes |  |  |  |  |  |  |  |  |  |  |  |  |  | 321 | 1.13 (0.62–2.06) | 0.682 |  |  |  |  |
| **Disease status** |  |  |  |  |  |  |  |  |  |  |  |  |  |  |  |  |  |  |  |  |
| relapsed |  |  |  |  |  |  |  |  |  |  |  |  |  |  |  |  |  | 229 | reference |  |
| primary refractory |  |  |  |  |  |  |  |  |  |  |  |  |  |  |  |  |  | 35 | 1.14 (0.66–1.98) | 0.630 |
| relapsed and refractory |  |  |  |  |  |  |  |  |  |  |  |  |  |  |  |  |  | 49 | 2.64 (1.79–3.90) | **< 0.001** |

*Results from Cox proportional hazard model*
