# Supplementary material for: Survival benefit of ixazomib, lenalidomide and dexamethasone (IRD) over lenalidomide and dexamethasone (Rd) in relapsed and refractory multiple myeloma patients in routine clinical practice
Source: BMC Cancer. 2021 Jan 15;21:73. doi: 10.1186/s12885-020-07732-1 (PMC7810195; doi:10.1186/s12885-020-07732-1)
Supplement: Supplementary file 3 — Additional file 3: Supplementary Table 3a. Association of ORR with selected variables. Supplementary Table 3b Association of ORR with selected variables in multivariable analysis – Paired analysis. [file 12885_2020_7732_MOESM3_ESM.zip › Supplementary 3aR3.docx]

**Supplementary table 3a** Association of ORR with selected variables

|  |  | **Logistic regression model** | | | | | | |  |
| --- | --- | --- | --- | --- | --- | --- | --- | --- | --- |
|  |  | **Univariable analysis** | | |  | **Multivariable analysis** | | |  |
|  |  | **N** | **Odds ratio (95% CI)** | **p-value** |  | **N** | **Odds ratio (95% CI)** | **p-value** |  |
| **Regimen** |  |  |  |  |  |  |  |  |  |
| IRD |  | 126 | reference | – |  | 117 | reference | – |  |
| RD |  | 216 | 1.38 (0.85–2.24) | 0,191 |  | 158 | 1.21 (0.67–2.20) | 0,527 |  |
| **Age (at treatment initiation)** |  |  |  |  |  |  |  |  |  |
| **≤ 65** |  | 136 | reference | – |  | 105 | reference | – |  |
| **66–75** |  | 147 | 1.52 (0.91–2.54) | 0,111 |  | 121 | 1.46 (0.73–2.95) | 0,286 |  |
| **> 75** |  | 59 | 0.75 (0.40–1.40) | 0,360 |  | 49 | 0.60 (0.22–1.68) | 0,334 |  |
| **Extramedullary mass** |  |  |  |  |  |  |  |  |  |
| **no** |  | 304 | reference | – |  | 247 | reference | – |  |
| **yes** |  | 32 | 1.14 (0.51–2.57) | 0,744 |  | 28 | 1.13 (0.44–2.90) | 0,797 |  |
| **ASCT in previous lines** |  |  |  |  |  |  |  |  |  |
| **no** |  | 171 | reference | – |  | 135 | reference | – |  |
| **yes** |  | 171 | 0.97 (0.62–1.54) | 0,907 |  | 140 | 0.48 (0.21–1.07) | 0,072 |  |
| **Previous treatment by PI** |  |  |  |  |  |  |  |  |  |
| **no** |  | 23 | reference | – |  | 19 | reference | – |  |
| **yes** |  | 319 | 0.96 (0.38–2.40) | 0,927 |  | 256 | 1.20 (0.41–3.49) | 0,744 |  |
| **Disease status** |  |  |  |  |  |  |  |  |  |
| **relapsed** |  | 228 | reference | – |  | 202 | reference | – |  |
| **primary refractory** |  | 35 | 0.32 (0.16–0.67) | **0,002** |  | 30 | 0.26 (0.10–0.65) | **0,004** |  |
| **relapsed and refractory** |  | 48 | 0.57 (0.30–1.10) | 0,092 |  | 43 | 0.60 (0.28–1.26) | 0,174 |  |
| **Lenalidomide dose (at treatment initiation)** |  |  |  |  |  |  |  |  |  |
| **≤ 10** |  | 41 | reference | – |  | 39 | reference | – |  |
| **11–20** |  | 58 | 2.23 (0.94–5.29) | 0,070 |  | 53 | 1.34 (0.51–3.55) | 0,550 |  |
| **> 20** |  | 204 | 1.70 (0.85–3.39) | 0,132 |  | 183 | 1.44 (0.65–3.18) | 0,371 |  |
| *Results from logistic regression model* |  |  |  |  |  |  |  |  |  |
|  |  |  |  |  |  |  |  |  |  |
