# Supplementary material for: Survival benefit of ixazomib, lenalidomide and dexamethasone (IRD) over lenalidomide and dexamethasone (Rd) in relapsed and refractory multiple myeloma patients in routine clinical practice
Source: BMC Cancer. 2021 Jan 15;21:73. doi: 10.1186/s12885-020-07732-1 (PMC7810195; doi:10.1186/s12885-020-07732-1)
Supplement: Supplementary file 3 — Additional file 3: Supplementary Table 3a. Association of ORR with selected variables. Supplementary Table 3b Association of ORR with selected variables in multivariable analysis – Paired analysis. [file 12885_2020_7732_MOESM3_ESM.zip › Supplementary 3bR3.docx]

**Supplementary table 3b** Association of ORR with selected variables in multivariable analysis – Paired analysis

|  |  | **Paired analysis - Regimen and selected variable** | | | | | | | | | | | | | | | | | | |
| --- | --- | --- | --- | --- | --- | --- | --- | --- | --- | --- | --- | --- | --- | --- | --- | --- | --- | --- | --- | --- |
| **Subgroup** |  | **N** | **Hazard ratio (95% CI)** | **p-value** |  | **N** | **Hazard ratio (95% CI)** | **p-value** |  | **N** | **Hazard ratio (95% CI)** | **p-value** |  | **N** | **Hazard ratio (95% CI)** | **p-value** |  | **N** | **Hazard ratio (95% CI)** | **p-value** |
| **Regimen** |  |  |  |  |  |  |  |  |  |  |  |  |  |  |  |  |  |  |  |  |
| RD |  | 126 | reference |  |  | 126 | reference |  |  | 126 | reference |  |  | 126 | reference |  |  | 119 | reference |  |
| IRD |  | 216 | 1.31 (0.80–2.15) | 0.280 |  | 210 | 1.32 (0.81–2.15) | 0.273 |  | 216 | 1.40 (0.86–2.30) | 0.177 |  | 216 | 1.39 (0.85–2.26) | 0.185 |  | 192 | 1.29 (0.77–2.16) | 0.337 |
| **Age (at treatment initiation)** |  |  |  |  |  |  |  |  |  |  |  |  |  |  |  |  |  |  |  |  |
| ≤ 65 |  | 136 | reference |  |  |  |  |  |  |  |  |  |  |  |  |  |  |  |  |  |
| 66–75 |  | 147 | 1.53 (0.91–2.57) | 0.105 |  |  |  |  |  |  |  |  |  |  |  |  |  |  |  |  |
| > 75 |  | 59 | 0.79 (0.42–1.50) | 0.477 |  |  |  |  |  |  |  |  |  |  |  |  |  |  |  |  |
| **Extramedullary mass** |  |  |  |  |  |  |  |  |  |  |  |  |  |  |  |  |  |  |  |  |
| no |  |  |  |  |  | 304 | reference |  |  |  |  |  |  |  |  |  |  |  |  |  |
| yes |  |  |  |  |  | 32 | 1.08 (0.48–2.45) | 0.851 |  |  |  |  |  |  |  |  |  |  |  |  |
| **ASCT in previous lines** |  |  |  |  |  |  |  |  |  |  |  |  |  |  |  |  |  |  |  |  |
| no |  |  |  |  |  |  |  |  |  | 171 | reference |  |  |  |  |  |  |  |  |  |
| yes |  |  |  |  |  |  |  |  |  | 171 | 0.92 (0.58–1.46) | 0.718 |  |  |  |  |  |  |  |  |
| **Previous treatment by PI** |  |  |  |  |  |  |  |  |  |  |  |  |  |  |  |  |  |  |  |  |
| no |  |  |  |  |  |  |  |  |  |  |  |  |  | 23 | reference |  |  |  |  |  |
| yes |  |  |  |  |  |  |  |  |  |  |  |  |  | 319 | 0.90 (0.36–2.26) | 0.816 |  |  |  |  |
| **Disease status** |  |  |  |  |  |  |  |  |  |  |  |  |  |  |  |  |  |  |  |  |
| relapsed |  |  |  |  |  |  |  |  |  |  |  |  |  |  |  |  |  | 228 | reference |  |
| primary refractory |  |  |  |  |  |  |  |  |  |  |  |  |  |  |  |  |  | 35 | 0.33 (0.16–0.69) | **0.003** |
| relapsed and refractory |  |  |  |  |  |  |  |  |  |  |  |  |  |  |  |  |  | 48 | 0.58 (0.30–1.12) | 0.107 |

*Results of logistic regression*
